# Therapy sculpts the complex interplay between cancer and the immune system during tumour evolution

**DOI:** 10.1186/s13073-022-01138-3

**Published:** 2022-12-07

**Authors:** Kerstin Thol, Piotr Pawlik, Nicholas McGranahan

**Affiliations:** 1 Cancer Research UK Lung Cancer Centre of Excellence, University College London Cancer Institute, London, UK; 2 Cancer Genome Evolution Research Group, University College London Cancer Institute, London, UK

**Keywords:** Tumour evolution, Immunotherapy, Immune evasion, Anti-cancer treatment

## Abstract

Cancer development is an evolutionary process. A key selection pressure is exerted by therapy, one of the few players in cancer evolution that can be controlled. As such, an understanding of how treatment acts to sculpt the tumour and its microenvironment and how this influences a tumour’s subsequent evolutionary trajectory is critical. In this review, we examine cancer evolution and intra-tumour heterogeneity in the context of therapy. We focus on how radiotherapy, chemotherapy and immunotherapy shape both tumour development and the environment in which tumours evolve and how resistance can develop or be selected for during treatment.

## Background

**Fig. 1:**
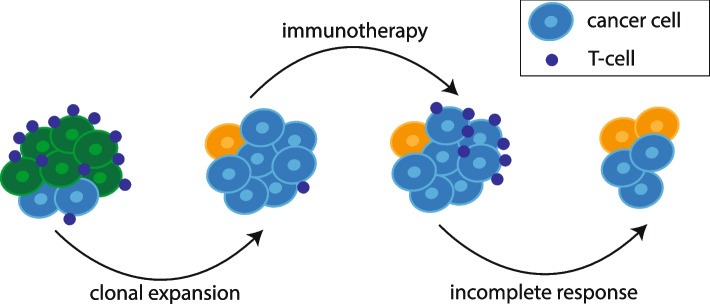
Clonal evolution is influenced by the immune microenvironment. Cancer cells which can evade attack by immune cells may undergo clonal expansion (blue subclone) while others perish (green subclone). Immunotherapy may lead to an increase of immune cells infiltrating the tumour and possibly tumour clearance but often the evolving tumour can acquire further immune evasive properties (orange subclone) resulting in an incomplete response to treatment

Tumours are subject to evolution by natural selection [1–4]. Heritable somatic mutations accumulate over time in the tumour genome. A subset of these mutations may confer a fitness advantage to the cancer cell. The fitness effects will be contingent upon the environment in which the cancer cell resides, which includes immune predation, therapy, and other factors [3, 5, 6]. Fitness translates into prevalence of a particular clone within the tumour, enabling it to outcompete other clones (Fig. 1).

Therapy may both directly and indirectly apply selection pressure and fuel tumour evolution [7]. Conventional cancer therapy, for example, chemotherapy and radiotherapy, aims to induce DNA damage, therefore leading to apoptosis and cancer cell death. Immunotherapy, on the other hand, aims to stimulate an anti-tumour immune response by facilitating the recruitment of active immune cells to the tumour to eliminate cancer cells. While the direct effects of different treatment modalities on tumours have been studied in depth, their impact on the tumour microenvironment and how evolutionary trajectories are shaped by treatment-directed immune modulation is less clear.

In this review, we explore the interplay between cancer evolution and the immune system in the context of treatment. We discuss how first-line therapy influences the natural immune response, how immunotherapy aims to overcome the challenges of immune escape, how to predict response to immunotherapy and, finally, how cancer vaccines aim to revolutionise personalised immunotherapy.

### The immune system and cancer-specific immune evasion mechanisms

The immune system represents the body's defence mechanism against disease, and consists of innate and adaptive components. While cancer cells can stimulate an immune response, there are also many ways a tumour may evade immune surveillance. In the following, we first describe the main functionality of the immune system and how cancer cells can be detected by the immune system. We then provide an overview of cancer cell-intrinsic and extrinsic immune evasion strategies, before discussing possible implications these may have on therapeutic approaches.

The innate immune system regulates the removal of newly encountered pathogens and cells which can be identified by the immune system as harmful or non-self by the expression of specific antigens. Meanwhile, the adaptive immune system is a more targeted system that triggers immune response to previously encountered antigens. T cells can recognise antigens presented by human leukocyte antigen (HLA) class I and II molecules which are expressed on the surface of cells, including professional antigen-presenting cells such as dendritic cells. Memory B cells, originating from a naïve lymphocyte which initially encountered an antigen, produce antibodies against previously encountered antigens.

Detecting cancer cells is a challenge for the immune system because—in contrast to exogenous pathogens—cancer cells abundantly express self-antigens. Self-antigens are expressed by all nucleated human cells and inhibit an autoimmune response. Nevertheless, most tumours are immunogenic to a certain extent (reviewed in [8]), but the degree of immunogenicity depends on many factors and varies between tumours. The overexpression of tumour-associated antigens [9] but also neoantigens—novel peptides produced as a consequence of mutations and structural rearrangements in cancer—can activate immune pathways. Neoantigens can be identified as ‘foreign' by the immune system when presented by the HLA system. For a neoantigen to elicit an immune response, it must be presented at the draining lymph node by professional antigen presenting cells, allowing a T cell to recognise the antigen, proliferate and leave the lymph node to infiltrate the tumour and kill cancerous cells. Thereby, an effective antitumor immune response is achieved due to the expansion of a subset of T cells against an identified threat and the subsequent elimination of the threat.

To evade an effective immune response, tumour cells have been shown to acquire various cell-intrinsic immune evasive strategies [10]. Immune checkpoint molecules on T cells and their ligands on cancer cells, such as programmed cell death protein 1 (PD-1) and programmed death-ligand 1 (PD-L1) prevent T cell activation when bound to each other. Blocking the interaction between these proteins and their cognate receptors on the immune cells is the foundation of immune-checkpoint blockade (ICB) drugs such as pembrolizumab.

An active immune response depends on the correct presentation of antigens derived from tumour cells to T cells, and impairment of this process constitutes immune evasion. It is becoming increasingly clear that antigen presentation disruption is one of the most frequent mechanisms of immune evasion in cancer [11]. For instance, loss-of-heterozygosity (LOH) of at least one of the *HLA* alleles is common in cancer [12] and leads to impaired presentation of cancer neoantigens. In treatment-naïve non-small cell lung cancer (NSCLC), this event occurred in ~40% of tumours, was often subclonal (i.e. present in only a subset of cancer cells), and developed later in tumour evolution [13]. In a pan-cancer study, LOH or homozygous deletions of *HLA* were seen in 18% (801 samples) of metastatic and 17% (325 samples) of primary tumour samples [14]. A study of 17 metastatic melanoma patients found that Beta-2 microglobulin (*B2M*), another essential component of the HLA class I antigen presentation machinery, was mutated, deleted or subject to LOH in 29% of patients whose tumours did not respond to immune checkpoint blocking therapy [15]. In this study, *B2M*-mutated subclones were selected early in tumour development. Furthermore, a signal of positive selection was observed for truncating mutations in *HLA-I* alleles in a pan-cancer analysis of 4514 metastatic and 1943 primary tumour samples [14]. In tumours with microsatellite instability (MSI), which are highly mutated and generally respond well to ICB, mutations in *HLA-B* were enriched compared to non-MSI tumours [16], possibly rendering these tumours resistant to ICB. In addition to genomic events hindering the correct presentation of neoantigens, epigenetic regulation of *HLA* is an added factor to consider. The expression of HLA molecules is epigenetically regulated by the acetyltransferase p300/CBP and NF-κB. These components can be activated by low doses of chemotherapy which leads to an upregulation of antigen presentation, thereby presenting a potential therapeutic target [17, 18]. A recent study by Dubrot et al. suggested a context-dependent role of *HLA* expression: in tumours where response is driven by T cells, expression of antigen presentation-related genes enhances the immune response, but if the response is driven primarily by natural killer (NK) cells, expression of *HLA* genes impairs the anti-tumour reaction by inhibiting the NK cells [10].

If a subset of cancerous cells lose their ability to present neoantigens effectively, this may have several implications in the clinical setting: immune checkpoint blocking agents or cancer vaccines which depend on the presence of immune cells in the TME or the presentation of neoantigens by cancer cells respectively may be less efficacious. Possibly, more aggressive subclones will be selected for as a consequence, which are then able to employ additional immune evasion strategies, further complicating their treatment.

In addition to the impairment of antigen presentation mechanisms, a reduction of the number of neoantigens expressed on the surface of cancer cells may facilitate immune evasion. Treatment naïve NSCLC tumours with high levels of immune infiltrate and with intact *HLA* alleles were shown to have a reduced expression of neoantigens compared to tumours with *HLA* LOH [19], suggesting that downregulation of neoantigen expression was selected for in response to an active immune response, even in a treatment naïve setting. In the same study, promoter hypermethylation of genes harbouring putative neoantigens was identified as a mechanism of neoantigen presentation disruption, emphasising that the epigenetic landscape may contribute to the immunogenicity of a tumour [19].

The anatomical location of a tumour and its underlying aetiology can also have an effect on the composition of the tumour microenvironment. Tumour mutational burden (TMB), the repertoire of neoepitopes, the immunogenicity of a tumour, and response to ICB are thought to be tightly linked [20–22]. TMB may be influenced by the mutational processes that were active during the tumour’s evolutionary history, including exposure to exogenous carcinogens [23]. However, TMB is by no means uniform across cancers from the same cancer types at the same anatomical location and of the same cell type of origin, and can vary greatly from tumour to tumour. Additionally, the accurate calculation and comparison of TMB depends on the applied sequencing technology [24, 25], source material, and bioinformatics processing pipeline [26]. The composition of different immune cells of a tumour also differs by location and are associated with progression [27]. Santegoets et al. showed that tumours of the cervix and the oropharynx, both induced by human papillomavirus (HPV) and of the same cellular origin, showed different immune cell abundances and compositions which resembled that of their normal tissues of origin [28]. The CD4:CD8 ratio in oropharyngeal cancer was 3-fold higher than that in cervical cancer, emphasising that anatomical location is an important factor for immunogenicity of a tumour and in consequence may—together with exposure to carcinogenic factors—shape the evolutionary trajectory of tumours.

Aneuploidy and somatic copy number aberrations (SCNAs), which may trigger endoplasmic reticulum (ER) stress, may influence the TME. ER stress is induced when proteins cannot be folded correctly, as is often the case in cancer. ER stress can lead to the phosphorylation of the Eukaryotic translation initiation factor 2 subunit 1 (eIF2a) which regulates immune related molecules, including PD-L1, and tumour infiltrating macrophages. Furthermore, after the induction of ER stress, Interleukin 6 (IL6) and Arginase 1 (ARg1) become activated which leads to increase in immunosuppression mediated by macrophages and dendritic cells [29, 30]. Additionally, chromosomal instability (CIN) can lead to the formation and subsequent rupture of micronuclei in the cytosol. The released double stranded DNA can be sensed by cGAS-STING pathway components. Under normal conditions, cGAS-STING activates canonical NF-κB signalling and downstream type 1 interferon signalling; however, in cells with high CIN, non-canonical NF-κB signalling was shown to be activated, which decreased type 1 interferon signalling and increased metastasis [30]. SCNAs and aneuploidy may also alter the TME by disrupting specific genes. Loss of chromosome arm 9p or parts thereof has been proposed to be used as an alternative to TMB or PD-L1 staining for ICB efficacy predictions [31, 32]. This is likely due to deletions encompassing the Interferon gene cluster, which are often found to be co-deleting the tumour suppressor locus *CDKN2A*, rendering cells less immunocompetent and more proliferative [33].

In summary, disruption of the antigen presentation machinery and somatic copy number aberrations are amongst a catalogue of different cancer cell-intrinsic mechanisms and conditions enabling immune evasion.

Cancer cell-extrinsic mechanisms may also contribute to an immune evasive phenotype. Regulatory T cells (Tregs) maintain self-tolerance to prevent autoimmunity of normal cells and have immune-suppressive functions in the cancer setting. Liu et al. showed that tumour cells expressed higher levels of Transforming growth factor β (TGF-β) than normal cells which converted CD4+CD25- T cells into Tregs and suppressed T cell proliferation [34]. Blocking CCR5, a chemokine receptor responsible for Treg recruitment to the tumour site, led to a depletion of Tregs and slowed down tumour growth in a mouse model [34, 35], indicating the involvement of Tregs in regulating the proliferative capacity of tumour cells. Furthermore, cancer-associated fibroblasts (CAFs), which are abundant in the stromal compartment of the tumour microenvironment, have been reported to have both tumour-inhibiting and tumour-facilitating [36] functions. CAFs may contribute to immune evasiveness of tumours by blocking effector T cell proliferation and promoting differentiation of CD4+CD25+ T lymphocytes into Tregs [37]. Antigen-presenting CAFs (apCAFs) have been proposed to be derived from the mesothelium and their presence in tumours of 33 pancreatic ductal adenocarcinoma patients was positively correlated with the presence of Tregs, leading to an inhibition of CD8 T cells [38]. This class of CAFs might be targetable by anti-mesothelin-antibody. The tumour stroma in the TME may furthermore promote immune evasive mechanisms by forming a barrier preventing the interaction of cancer cells and lymphocytes [39] and increased physical contact between stromal and immune cells may also be observed in immune cold tumours [40]. To summarise, the composition of the TME can have effects on immune evasive properties of the tumour which can potentially be targeted by less well established immune-promoting therapies.

Tumour evolution is a major challenge for cancer therapy. In a sub-cohort of primary metastatic colorectal cancers with a higher percentage of cytotoxic T cells than the cohort mean, primary tumours which metastasized had a lower abundance of innate immune cells and memory T cells compared to primary tumours which did not metastasize [41]. When adaptive immune cells were already present in the primary tumour, recurrence and metastasis were less likely in this study. Waterman and colleagues showed that levels of T cell infiltration were reduced at the time of a second recurrence compared to the primary tumour [42]. In contrast, in early relapsed hepatocellular carcinoma, an increase of cytotoxic T cells was reported in relapsed tumour samples; however, these T cells exhibited dysfunctional cytotoxicity and had a reduced clonal expansion capacity compared to T cells in paired primary samples [43], indicative of an immune evasive phenotype.

Tumour immunity depends on a complex array of factors, and immune-modulatory mechanisms can change over time and space within a tumour, often leading to immune evasion. Both tumour cell intrinsic (e.g. disruption of the antigen-presentation machinery) and extrinsic properties (e.g. composition of the stromal compartment and presence of Tregs) play a role in immune surveillance and impairment thereof. Environmental influences, such as anti-cancer therapy, can further apply selective pressure on the immune system and cancer cells.

### Conventional cancer treatment influences the immune system

Emerging data suggest cancer treatment influences the immune system's response to a tumour and may thereby also alter its evolutionary trajectory [44–46]. Chemotherapy is often the first-line therapy for many different types of cancer. Platinum-based chemotherapy induces DNA adducts in dividing cells. The formation of such structures between platinum and the DNA which can lead to inter- and intrastrand cross-linking of DNA strands, typically results in apoptosis. This mechanism is most effective in rapidly dividing cells such as tumour cell populations and cells comprising the bone marrow. As lymphocytes are produced in the bone marrow, immunosuppression is a common side-effect of chemotherapy [47–49].

Chemotherapy can also have immunostimulatory effects. Jimenez-Sanchez and colleagues reported an increase in the expression of immune-related hallmark gene sets and immune cells in post-chemotherapy high-grade serous ovarian cancer (HGSOC), compared to site-matched untreated HGSOC tumour samples [46]. They observed an increase in natural killer (NK) cell count following neoadjuvant chemotherapy and were able to confirm a similar effect in a preclinical mouse model following platinum chemotherapy. The immunomodulating effects of chemotherapy would have been missed without paired samples, emphasising the importance of longitudinal studies of tumour evolution [46]. Another immunostimulatory effect was reported by Homma et al., where gemcitabine-based chemotherapy led to a depletion of Tregs in peripheral blood samples in pancreatic cancer [50]. These studies highlight that chemotherapy does not only impact cancerous cells but also affects the TME composition, thereby shifting cancer evolutionary dynamics.

Somatic mutations in a tumour genome are the result of a mutagen and impaired repair of the induced DNA damage. Conceivably, therefore, one might expect to find an increased neoantigen burden in tumours treated with chemotherapy, especially in those tumours which harbour DNA repair mechanism defects. Different chemotherapeutic agents, but especially cisplatin, have been shown to induce single base substitutions and short insertions and deletions [51]. In ovarian cancer, neoantigen burden was indeed elevated in relapsed tumours previously treated with chemotherapy. However, only a minority of neoantigens (5%) could be directly attributed to mutations derived from chemotherapy (as measured by mutations attributed to a chemotherapy mutational signature) and the majority stemmed from mutations associated with BRCA deficiency [52], possibly indicating that indirect mutagenesis by impairment of DNA repair mechanisms has a stronger effect on neoantigen generation than DNA damage induced by chemotherapy directly. More evidence for indirect treatment-induced mutagenesis was presented by Russo et al. who showed that BRAF and EGFR inhibition in colorectal cancer cell lines down-regulated high-fidelity DNA repair pathways and up-regulated error-prone DNA repair pathways leading to increased DNA damage. This effect ceased after drug treatment and was possibly activated by the induction of cell stress-related mTOR signalling or reactive oxygen species; the authors speculated that the effect was caused by the activation of an ancestral programme which accelerates evolution in unicellular organisms under stress [53, 54]. Potential synergistic effects of systemic therapy mediated DNA damage and targeted therapies have furthermore been shown with gamma-secretase inhibitors (GSI) and chemotherapy. GSIs inhibit the Notch signalling pathway which is commonly upregulated in cancer. DNA damage can activate the Notch-1 intracellular domain and subsequent blocking of Notch sensitised cancerous cells to chemotherapy [55]. However, reports in cell lines have also shown the impairment of apoptosis following dual treatment with GSIs and chemotherapy [56]. Additionally, Notch signalling regulates Tregs, whereby blocking of the Notch pathway results in greater abundance of Tregs, which may have an immunosuppressive function [57]. Therefore, circumstances in which GSIs in combination with chemotherapy are advantageous to the patient need to be carefully assessed.

**Fig. 2:**
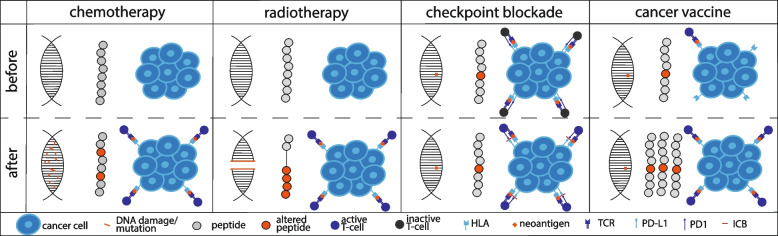
Therapy induced immunogenicity. Chemotherapy and radiotherapy may lead to the generation of neoantigens via DNA damage which may increase the immunogenicity of a cell. Checkpoint blockage and cancer vaccines do not alter the DNA but may lead to the activation of T cells and to increased neoantigen presentation to T cells respectively

Increased mutagenesis following drug treatment could have different effects on the evolution of tumour cells: firstly, increased mutation acquisition may increase the likelihood of a cancer cell harbouring a mutation associated with higher fitness to the changing environment; and secondly, an increased mutation burden may be associated with additional neoantigens which can trigger the natural immune response and could also be exploited therapeutically (Fig. 2).

Similarly to chemotherapy, radiation therapy (RT) can have anti- and pro-immunogenic effects. RT causes lesions of the DNA’s bases or sugar-phosphate backbone inducing single or double strand breaks usually activating apoptotic pathways. Lymphocytes are radiation sensitive, but radiation induced DNA damage and cell killing can result in an increase of macrophages, T cells and the activation of dendritic cells within the field of radiation [58–61].

In cancer cell lines, genes harbouring an immunogenic mutation were upregulated following radiotherapy [62]. *HLA* expression was upregulated in a melanoma cell line following radiation in a dose dependent manner due to an increased peptide pool following DNA damage [62, 63]. Radiotherapy also induces insertion-deletion mutations [64, 65] which could in theory generate neoantigens indicating the possibility for synergistic effects of radiotherapy and immunotherapy (Fig. 2). Turajlic et al. found that indel mutation derived neoantigens yielded more high-affinity binders than single nucleotide variant (SNV) mutations and frameshift indel burden was associated with checkpoint inhibitor response, especially for clonal frameshift mutations [66].

In an ovalbumin-expressing thymoma mouse model, radiotherapy combined with an mRNA vaccine against ovalbumin slowed primary tumour growth significantly more than RT or vaccination by itself. Tumour growth of the secondary tumour which was shielded from radiation was also slower in the group receiving RT and the mRNA vaccine compared to the vaccine alone or a non-treated control group [67]. Furthermore, anti-CTLA4, another ICB agent, and RT enhanced CD8+ T cell infiltration in vivo and tumour growth could be controlled more effectively using both treatments than each treatment alone by inducing ferroptosis—a type of programmed cell death [68]. One potential mechanism for the synergistic effects of RT and immunotherapy may be a radiotherapy mediated upregulation of tumour-associated antigens potentiating an immune response. The immune response may also extend to the non-irradiated tumour as T cells may already be primed to detect clonal antigens which might be shared by the secondary tumour. More evidence for the tumour evolution modulatory effects of radiotherapy in combination with immunotherapy affecting distant metastases outside of the field of radiation was provided by Ji and colleagues. In a colorectal cancer mouse model, the combination of RT and anti-CTLA4+PD1 increased tumour shrinkage in non-irradiated liver metastases in comparison to RT-IgG or RT+anti-CTLA4. This combination furthermore led to an increase of CD8+ T cells and a decrease of Tregs in liver metastases [69]. Possibly, metastases with higher mutational burden are more prone to undergo abscopal effects as a radiotherapy-induced immune response could have a stronger effect in tumours with higher neoantigen burden, therefore analysing differing mutational backgrounds and their influence on abscopal effects could be of interest.

A synergistic effect was also seen with radiotherapy and interleukin-15; here, the dosage of radiotherapy was important, as the effect was only seen at a lower dosage given in several fractions (dosage of 8GyX3) but not at a higher dosage given in a single fraction (20GyX1) [70]. This combination treatment also increased the dendritic cell infiltrate within tumours of the used mouse model, indicating the possibility to exploit this effect in tumours with impaired antigen presentation [70]. The immunomodulating effects of radiotherapy were proposed to be considered for more accurate radiotherapy treatment planning in a theoretical framework by Montaseri et al. The size of tumours, their levels of vascularization, hypoxia and necrosis and how these factors influence the level of immune infiltration and radiotherapy sensitivity can be exploited to predict response to radiation therapy and synergy between radiation and immunotherapy [71].

Surgery of any type, including removal of tumours, causes a trauma to cellular tissues. The inflammatory events triggered by surgery often have pro-tumorigenic qualities. For example, neutrophil extracellular traps (web-like, extracellular DNA structures decorated with proteins [72]) enhance the metastatic potential of cancer cells [73], NK cells lose their cytotoxic potential [74] and T cells lose their ability to suppress pre-existing, distant micrometastases [75]. Additionally, surgical resection of the tumour draining lymph nodes may deplete a naturally occurring or ICB induced immune response towards the tumour. Lymph nodes are the hub for T cells and dendritic cells. In a mouse model of head and neck cancer, removal of the lymph nodes led to a worse outcome in combination with ICB [76]. However, lymph nodes are also the site from which metastasis often disseminate to distant organs [77]; therefore, sparing lymph nodes may not be advantageous. Saddawi-Konefka et al. propose to administer ICB before ablating lymph nodes to effectively elicit an immune response and to minimise the risk of metastasis. Ideally, we would be able to predict which tumours are less likely to metastasise and spare tumour draining lymph nodes in these patients to ensure the best possible immune reaction.

In summary, it is becoming increasingly clear that therapies often have both pro- and anti-tumour effects (Table 1) and that chemo- and radiotherapy can have immunomodulating effects, both by directly inducing mutations and more generally by increasing the likelihood of generating neoantigens due to DNA damage (Fig. 2). However, neoantigens generated by cytotoxic treatment associated mutations will be subclonal unless bottle-necking occurs and therefore may not result in a durable immune response without additional immune activation. Such subclonal neoantigens may, furthermore, be hindered to elicit a strong immune response due to immunodominance towards the earlier clonal neoantigens [78].

**Table 1:** Interactions between selected treatment modalities and the immune system

Therapy type	Anti-tumour immune interactions	Pro-tumour immune interactions
Chemotherapy	Immunostimulation [46]; Treg depletion [50]; Increased mutational burden (e.g. by Cisplatin [51]); Stress-induced accelerated evolution [53, 54]^a^	Immunosuppression (platinum adducts cause apoptosis of lymphocytes [47–49]); Stress-induced accelerated evolution [53, 54]^a^
Surgery		Surgery promotes metastasis and is immunosuppressive [73–75]; Removal of lymph nodes removes anti-tumour lymphocytes [76]
Radiotherapy	Radiation-mediated cell-killing increases activation of macrophages, T cells and dendritic cells [58–61] and increases HLA expression [62, 63] and neoantigen load [64, 65]; abscopal and synergistic effects with anti-CTLA4+PD1 agents via activated lymphocytes [68]; radiation + IL-15 recruits dendritic cells [70]	DNA damage of lymphocytes [58]
Targeted therapies	BRAF inhibitors reverse BRAF-induced HLA internalisation [79]	MAPK inhibitors cause resistance to ICB [80]; EGFR inhibitors + PRC2 downregulates HLA I and B2M [81]
ICB	ICB + epigenetic therapy promotes anti-tumour TME [82–84]	Hyperprogression [85, 86]

^a^Acquisition of new mutations might both be beneficial and detrimental to the patient, depending on the context

### The interplay of immunotherapy and cancer evolution

Immune checkpoint blockade therapy aims to harness the immune system and is both affected by and shapes tumour evolution. ICB has proven to be effective only in a subset of tumour types and a subset of patients. Here, we discuss the main determinants of ICB resistance and their role in predicting response to ICB, suggest how such biomarkers could be improved in the future as well as how resistance to immunotherapy is acquired.

#### Predicting immunotherapy response

**Fig. 3:**
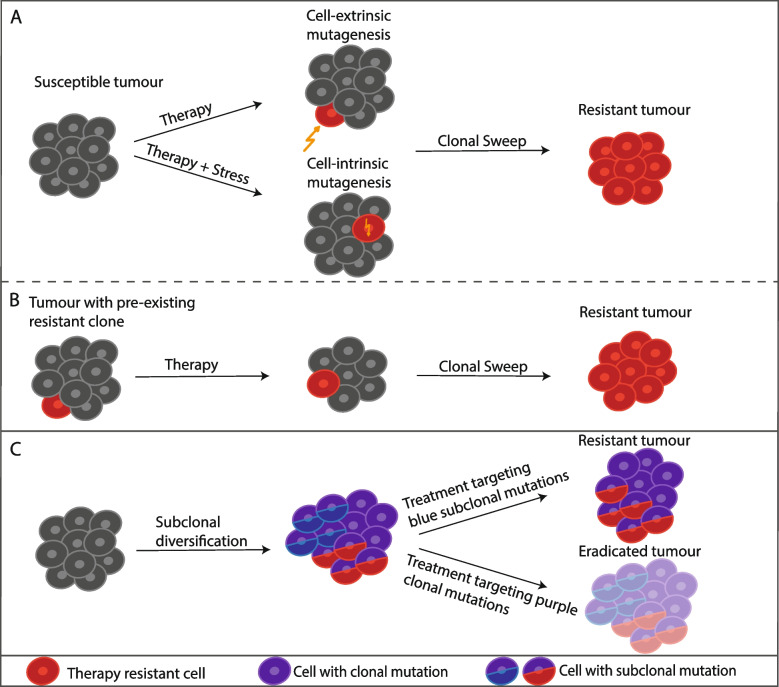
Resistance and therapy. **A** Even fully clonal tumours can develop resistant subclones during therapy. New mutations can be caused by external factors, such as radiation, or by internal reprogramming, such as upregulation of error-prone DNA polymerase and downregulation of DNA repair mechanisms. **B** Tumours with minor resistant subclones are likely to re-emerge after therapy. To avoid this, tumours should undergo detailed characterisation, for example by multisampling or using single-cell analysis. **C** In tumours harbouring multiple subclones, treatments—such as vaccines—should target truncal (i.e. present in every tumour cell) targets

ICB often triggers systemic inflammatory side effects [87, 88] and many tumours develop resistance during therapy or do not respond at all. In certain cases, not only do patients fail to respond to ICB, but the disease accelerates after treatment, a phenomenon known as hyperprogression [85, 86]. These factors, coupled with the high cost of ICB therapies, have spurred a significant effort to identify factors that predict response to ICB or the acquisition of resistance (Fig. 3).

The presence of the PD-L1 protein, detected by immunohistochemistry staining, has been proposed as one such factor and is one of the three United States Food and Drug Administration (FDA)-approved biomarkers of response. The remaining two FDA approved biomarkers are microsatellite instability (MSI) and tumour mutational burden, both of which primarily separate responders from non-responders based on the presence of a high number of mutations and in consequence high levels of neoantigens. However, the three biomarkers are imperfect. Moreover, tumours predicted to respond based on PD-L1 staining are seldom predicted to respond based on TMB analysis, suggesting that there are multiple factors determining response and currently approved approaches do not cover the whole spectrum of response or resistance determinants [89, 90].

The need for multifactorial analysis of response has been conceptualised as the ‘cancer immunogram’ [91], which postulates that T cells are the ultimate effectors of antitumor activity, but multiple factors influence their efficiency. For example, a tumour might express high levels of PD-L1, but lack tumour-infiltrating lymphocytes (TILs), thus making ICB ineffective without any other means of attracting T cells to the tumour. This means that predicting a response along a singular axis (e.g. by considering only PD-L1 expression) might be insufficient, and all parameters influencing the anti-cancer response should be taken into account. Emerging ICB prediction models try to capture the complex interactions between cancer and immune cells by evaluating signals coming from both the tumour and the tumour microenvironment [92] and include novel prediction features such as LOH of *HLA* [93], levels of T cell dysfunction and infiltration [94] or expression of inflammation-associated genes [95, 96].

Tumours are often composed of a myriad of distinct cancer subclones, which may harbour distinct sets of mutations affecting sensitivity or resistance to therapy. Emerging work suggests that clonal neoantigens may elicit a more sustained and effective immune response [97]. Less heterogeneous tumours derived by single-cell cloning of cells from a UVB radiation-exposed melanoma cell line, which had a lower number of tumour subclones than their parental cells, were shown to exhibit increased immunogenicity and slower tumour growth [98]. Additionally, an increased fraction of clonal neoantigens was associated with smaller tumour volume in mismatch repair-deficient colorectal cells with an elevated mutational burden but not in mismatch repair competent cells, possibly because of an activated immune response [99]. The presence of high clonal neoantigen burden in tumours with low neoantigen intra-tumour heterogeneity was furthermore shown to be associated with longer progression free survival in anti-PD-1 treated NSCLC and also associated with longer overall survival in anti-CTLA4 treated melanoma [21]. Subclonal mutations induced by alkylating agents in 3 patients also treated with immune checkpoint inhibitors did not drive durable responses possibly because not all tumour cells would be detected by T cells [21].

Tumour heterogeneity may also directly influence the immune microenvironment. Lee et al. described four lung tumours in which multiregional analysis found profound discordance in predicted anti-PD-1 response within different regions of the same tumour [100]. Conceivably, in such cases, treating patients with ICB could lead to a clonal sweep and domination of ICB-resistant clones. Furthermore, Hong et al. found a significant difference in PD-L1 expression at different biopsy sites in 398 NSCLC patients. Adrenal and liver metastases showed high expression of PD-L1, with brain and bone sites at the other end of the spectrum—the latter likely due to a specific immune environment both in the brain and bones (immune privilege and low levels of cytotoxic cells respectively [101]). In-depth studies of intra-tumour heterogeneity are required to establish if this state is the effect of ongoing selection in metastatic sites or a result of pre-metastatic evolution in primary sites.

#### Acquisition of immunotherapy resistance

Methods described in the previous section address the problem of primary resistance, i.e. whether the patient will respond to therapy or not, based on mutations already present at the time of diagnosis or sampling. However, resistance can also be acquired after initiation of therapy via mutagenesis induced by treatment, pre-existing mutations and genomic aberrations providing cancer cells with a selective advantage under therapeutic pressure, or via non-genetic mechanisms, for example, due to epigenetic changes and cellular plasticity [19, 81, 102].

Temporal changes in PD-L1 expression illustrate the dynamic nature of tumour-immune system interactions. A study by Hong et al. analysed 112 cancer patients for which PD-L1 expression was assessed at two different time points during the disease course, of which 77 received treatment (radiotherapy, surgery, chemotherapy, EGFR or ALK tyrosine kinase inhibitor or ICB) between biopsies. Patients who received ICB or another treatment combined with ICB displayed a significant decrease in PD-L1 expression, suggesting either an ongoing evolution, reprogramming of cancer cells to suppress the immune response [101], or the expansion of a minor ICB resistant subclone under the selective pressure of treatment.

Zaretsky and colleagues studied four melanoma patients treated with pembrolizumab who progressed despite initial response; their analysis shows how therapeutic pressure can lead to immune escape and influences the fitness effects of somatic mutations in certain genes [103]. One patient’s tumour displayed a homozygous loss-of-function mutation in *B2M*, resulting in impaired antigen presentation. This mutation was only found in a relapse biopsy and not in the pre-treatment sample, suggesting that it was either acquired after treatment, or it was present in a minor subclone not detected by the single-sample biopsy. Similarly, two other patients’ tumours showed relapse-specific loss-of-function of JAK1/2 (via homozygous mutation and LOH), which likely impaired response to interferon-γ signalling. In a homeostatic setting, interferon-γ inhibits growth, but also induces PD-L1 expression in cancer cells to inactivate T cells. After the introduction of pembrolizumab, however, this interferon-γ mediated PD-L1 induction mechanism ceased to be relevant and inactivating interferon-γ became beneficial for cancer cells [103].

Recent evidence points both to the synergistic effects and induction of cross-resistance between ICB and other types of therapies. Bradley et al. report internalisation of HLA-I induced by oncogenic BRAF^V600E^, reverted by treatment with MAPK inhibitors [79, 104], suggesting that the *BRAF* oncogene has immunomodulatory effects. In line with this evidence, combination of ICB with MEK and BRAF inhibitors improved response in murine models of melanoma. A study by Haas et al. shows how cross-resistance between inhibitors and ICB might arise. Here, melanoma *Braf*^*V600E*^ cell lines were used to create MAPK-inhibitor resistant tumours (by exposure to RAFi or RAFi/MEKi), in immunocompromised mice which were compared to tumours derived from MAPK-inhibitor naïve cells. After cell transplantation, both naïve and resistant tumours were treated with ICB, and only the non-MAPKi-resistant tumours showed a response. The resistant samples displayed an evasive tumour microenvironment characterised by reduced infiltration of functionally impaired T cells, an increase of myeloid cells and a decrease of CD103+ dendritic cells. Since the genetic makeup of the founder cells was known, the authors were able to conclude that ICB therapy resistance was not the result of a pre-existing therapy-resistant clone but arose under the MAPK-inhibitor treatment and its likely cause is a shift into a more immunosuppressive microenvironment. The authors suggest that immunotherapy as a first-line treatment could possibly prevent resistance from occurring. They also postulate that activating dendritic cells in resistant tumours with poly(I:C) therapy or focal radiotherapy could possibly re-sensitise tumours to immunotherapy when cross-resistance arises [80]. Another example of cross-resistance and epigenetic-mediated resistance has been shown by Burr and colleagues in a study of three lung adenocarcinoma tumours displaying a neuroendocrine transformation to small cell lung cancer under EGFR inhibition treatment with Erlotinib (a tyrosine kinase inhibitor). HLA class I components were downregulated in the treated tumours, specifically HLA I heavy chain and B2M, rendering the tumours resistant to ICB without any prior exposure to this type of treatment. These changes were mediated via Polycomb repressive complex 2 (PRC2) and were reversible upon inhibition of PRC2 [81]. Epigenetic-mediated resistance can furthermore be driven by mutations in *SETDB1,* a histone methyltransferase. Experimental knockout of *SETDB1* in mouse models increased sensitivity to ICB [105] and loss-of-function mutations in this gene in the A549 lung adenocarcinoma cell line resulted in impaired proliferation and decreased migration potential [106]. Similarly, Pan et al. and Miao et al. show that the SWI/SNF complex can contribute to immunosuppression via interferon-related genes and the inactivation of this chromatin remodelling complex leads to a better response to ICB [107, 108].

Another successful treatment modality is adoptive cell transfer (ACT), of which anti-CD19 CAR-T therapy is the most representative example. For this type of immunotherapy, the patient’s own T cells are modified to recognise new targets—for example the CD19 protein commonly expressed on B cells [109]. These therapies proved to be very efficient (e.g. 81% remission rate at 3 months [110]) but also susceptible to the development of resistance. Rosenthal et al. report that 17% of patients (in a cohort of 501 patients) had CD19 negative blasts (i.e. tumour-precursor cells) before the start of therapy, and these populations could lead to relapse after a successful elimination of CD19 positive clones with CAR-T therapy [111]. A few mechanisms of CAR-T resistance have been described, such as alternative splicing (which excludes the CAR-T binding epitope), hemizygous deletions of the CD19 locus, frameshift mutations [112] and lineage switching [113].

Difficulties in predicting response to immunotherapy and the acquisition of resistance are likely in large part driven by the heterogeneity of tumours. In some cases, a resistant clone already exists within the cancer cell population, potentially below the threshold of detection or in regions of a tumour not sampled during a biopsy. This risk can be mitigated by extensive sampling and by designing therapies against clonal targets. In other cases, therapy causes or accelerates the creation of resistant clones, a process which itself might depend on the tumour’s mutational background (i.e. faulty DNA repair pathways) and TME. These factors, as well as multiple underpinnings of anti-tumour response pose a challenge to response prediction. To address this issue, new models should be comprehensive and evolution-aware, but also take full advantage of the high-quality data obtained with emerging bioinformatics tools [114]. 

### Personalised vaccine approaches and combination treatment strategies

While vaccines have traditionally been used in a preventative context for infectious diseases, the notion that these agents could be harnessed in cancer treatment to elicit and amplify immune responses towards antigens has long been recognised. However, early studies seeking to exploit vaccines engendered scepticism; poor immunogenicity and, in particular, lack of tumour specificity are significant hurdles for successful vaccine approaches. More recently, personalised vaccine approaches based on tumour specific neoantigens have been proposed [115]. As described previously, in ‘immune cold' tumours, where immune cells are either absent or dysfunctional, immune checkpoint blockade is often not beneficial. Thus, increasing the efficacy of neoantigens using neoantigen vaccines predicted to bind the remaining HLA alleles could potentiate an immune response to a certain degree which can furthermore synergize with other immune-stimulating therapeutics.

In a promising study, personal neoantigen vaccines targeting 13-20 immunising long peptides were trialled in 6 melanoma patients [116]. Four of the patients remained recurrence-free at a median follow up of 25 months. Two patients progressed but showed complete radiographic response after pembrolizumab treatment. T cell lines against predicted epitopes arising from the vaccines showed especially high activity for epitopes arising from novel open reading frames. Vaccinated patients in the study by Ott et al. as well as in a study by Keskin et al., showed elevated levels of PD-1 expression, possibly as an immune evasion mechanism of cells which were not removed by a vaccine-mediated immune response [116, 117]. Several reports have shown the synergistic effects of personalised neoantigen vaccines and PD-1/PD-L1 blockade [118, 119]. Vaccine mediated T cells may be activated by PD-1/PD-L1 blockade, reversing the effects of PD-1 upregulation as an immune evasion strategy following vaccination.

The long-term effects of neoantigen vaccines were shown in metastatic melanoma where NeoVax personalised neoantigen vaccine-induced T cells detectable in blood were still responsive to immunising peptides up to 4.5 years after initial vaccination [119, 120]. Nevertheless, as the tumour evolves, cancer cells not previously eliminated—possibly due to a lack of vaccine targeted neoantigens or through other escape mechanisms—may continue to proliferate without being affected by vaccine-induced T cells.

Non-personalised vaccines (‘off-the-shelf’), designed to immunise against neoantigens frequently shared by several patients, have the potential to lower the costs of vaccines and make them more readily available. In MSI- and TMB-high cancers, an increased load of shared frameshift mutations was detected which could be targeted with ‘off-the-shelf’ vaccines in combination with anti-PD-1 ICB [121]. However, for tumours with a low mutation burden, such as glioma, an approach only targeting neoantigens might not be beneficial. For these tumours, it has been proposed to make use of not only somatic mutation derived neoantigens but also of HLA-presented peptides which are not derived from a somatic mutation but are either tumour-exclusive or overexpressed in the tumour. Both such antigens could be targeted with two separate vaccines; one highly personalised neoantigen vaccine and one for a general library of tumour-specific antigens which were not derived from somatic mutations and were selected for their immunogenicity [122]. Cancer treatment should take into account the heterogeneous nature of tumours. In this context, poly-epitope vaccines could be designed to target multiple epitopes simultaneously, either targeting multiple truncal mutations or a combination of clonal and subclonal epitopes. When in a study by Li et al. a polyepitope vaccine was applied to a patient with a metastatic neuroendocrine tumour, out of the targeted 13 neoantigens, three elicited an immune response, emphasising the need for accurate neoantigen prediction methods [123]. However, this study did not evaluate the heterogeneity of the tumour and did not compare the results to a single-epitope vaccine. The shared neoantigens in MSI-high tumours in the study by Roudko et al. [84] were predominantly subclonal but within each patient at least one clonal neoantigen was also detected, suggesting that in this study a wide variety of subpopulations of cells was effectively targeted.

Despite these advances in the development of cancer vaccines, this therapeutic strategy also faces several challenges [124]. Vaccine mediated T cells need to be able to infiltrate the tumour [125] and have cytotoxic activity [117]. Furthermore, for cancer vaccines to be effective, cancer cells need to be able to present neoantigens on their surface [126]—requirements which can be circumvented by known immune evasion mechanisms.

To evaluate additional approaches for overcoming the challenges of treatment resistance, combination treatments of checkpoint inhibitors with additional treatment regimens have been considered. For instance, to create a more immune-permissive tumour microenvironment the combination of anti-PD-1/PD-L1 therapy with epigenetic therapy has been explored [127, 128]. In a study by Knox and colleagues the combination of the histone deacetylase inhibitor HDAC6i—which was previously shown to decrease the expression of PD-L1 [129]—with anti-PD-1 antibodies, decreased tumour growth in a mouse melanoma model by shifting the TME phenotype from ‘cold’ to ‘hot’. This effect was achieved by reducing the expression of TGF-β and a decreased presence of immune-suppressive M2 macrophages in the TME [127].

ICB response can also be enhanced by exploiting viral mimicry. Inhibition of DNA methyltransferase (DNMTs) triggers the expression of endogenous retroviruses which results in an antiviral response against the affected cells via activation of interferon signalling [82]. Histone deacetylases (HDAC) and DNMTs can be inhibited jointly using combination epigenetic therapy. Topper and colleagues sought to optimise this combination by inhibiting both enzymes in tandem using NSCLC cell lines. The combination of azacitidine (a DNMTi and cytotoxic agent) and HDACi reduced MYC signalling leading to proliferative arrests of NSCLC cells. Application of this combination treatment to a NSCLC mouse model resulted in reduced tumour growth and a lower frequency of metastasis, possibly achieved by an increase in CD8+ T cell count. Furthermore, treatment over a period of 3 months resulted in a downregulation of T cell exhaustion associated genes and induction of memory-associated genes, indicating durable long-term responses and possibly a strategy for overcoming T cell exhaustion [83]. In this study, the addition of anti-PD1 treatment had no additional therapeutic effect, but Ghoneim et al. (2017) reported enhanced ICB efficacy upon methylation inhibition caused by reversing CD8+ T cell exhaustion [84]. Combination treatment of pembrolizumab, guadecitabine (DNMTi) and mocetinostat (HDACi) is currently being tested in a clinical trial (NCT03220477).

As mentioned above, in addition to PD-1/PD-L1 and CTLA-4 several other immune checkpoint molecules play a similar role in activating and enabling immune response. Multiple trials are currently evaluating alternative ICB targets such as TIM-3, LAG-3, TIGIT and VISTA. Applying ICB based on a combination of inhibitors might not only result in a more robust response to ICB but also help to target minor resistant clones, by bypassing clone-specific resistance mechanisms.

In addition to treating cancer after the disease arises, preventative measures targeting tumours before they arise or before they evolve metastatic or resistant would be of great clinical benefit. This strategy has previously proven successful for virus-induced cancers such as HPV induced cervical cancer [130]. Targeting tumorigenic mutations before they occur, such as somatic *KRAS* or *EGFR* mutations, could be another avenue for preventative vaccination by priming the immune system for predicted epitopes of these mutations. A vaccine using peptides computationally predicted to target neoantigens derived from mutant *KRAS* elicited an immune response in a mouse model of *KRAS*-driven lung cancer in which mice were vaccinated prior to activating the mutant *KRAS* gene [131]. This strategy however would only be beneficial if these mutations were tumour specific [132, 133]. Mathematical modelling efforts assessing how many cytotoxic T lymphocytes would be needed to eliminate a tumour before clinical detection showed that a population of 3% or less could eradicate a developing tumour population [134]. Hartmeier et al. identified mutations in *KRAS*, *PIK3CA* and *EGFR* which were present in 45% of examined tumours, to study whether mutations present in many different cancer types could be utilised for widely useful cancer vaccines. Their estimations predict that such vaccines would only be applicable to ~0.3% of the population, based on a study of ~63,000 tumours, due to HLA diversity [135]. This study emphasises the difficulties of making widely effective preventative cancer vaccines a reality. If such vaccines ever become the main-stream, as they have for cervical cancer, the field of cancer evolution will change drastically. Focus will likely shift towards the personalised treatment of tumours with rare driver mutations and highly aggressive tumours which have evolved instantly to escape an initial immune response. Therefore, past and present improvements of personalised medicine will be profoundly useful.

While cancer vaccines are a promising strategy to activate the body’s own instruments to counteract the formation of a tumour, some challenges still lay ahead of their routine use. Generalised cancer vaccines may be easier to access in the clinical setting, but personalised vaccines and other personalised treatment strategies likely have greater efficacy for more cancer types.

## Conclusions

Evolution is frequently erroneously depicted as a linear increase of complexity—as illustrated in the ‘March of Progress' cartoon [136]. Cancer is often viewed in a similar manner—as a linear process accumulating increasing genomic and transcriptomic complexity, culminating in metastasis. In reality, neither species nor tumour evolution can be truly captured by a linear trajectory [137–139]. Cancer evolution is a complex process whereby heterogeneity is the norm rather than the exception. The immune system and treatment selection pressures play a key role in sculpting this complex process. Conventional anti-cancer therapy can elicit an immune response, immune checkpoint blockade is used to activate tumour infiltrating T cells and cancer vaccines are employed to enhance the detectability of a tumour by the immune system. However, the heterogeneity of tumours allows subsets of cells to evade attack by the immune system in many cases.

If we are to successfully treat this devastating disease, we must leverage an active immune system to help tackle the inevitable evolution. An increasing body of knowledge, sequencing technologies and better diagnostic tools are needed to help elucidate more clinically relevant targets, helping to treat cancer before immune evasion and resistance mechanisms occur. Tailoring treatment to a tumour’s individual genomic landscape or targeting alternative pathways activating an immune response could be beneficial for treating an evolving tumour. Targetting clonal neoantigens as well as finding suitable and effective treatment strategies early on in the evolutionary timeline of a tumour could drive durable immune responses.

## Data Availability

Not applicable

## Abbreviations

ACT: Adoptive cell transfer
apCAFs: Antigen-presenting cancer-associated fibroblasts
B2M: Beta-2 microglobulin
CAFs: Cancer-associated fibroblasts
CIN: Chromosomal Instability
DNMT: DNA methyltransferase
ER: Endoplasmic reticulum
FDA: The United States Food and Drug Administration
HDAC: Histone deacetylase
HLA: Human leukocyte antigen
HPV: Human papillomavirus
GSI: Gamma-secretase inhibitors
ICB: Immune checkpoint blockade
LOH: Loss of heterozygosity
MSI: Microsatellite instability
NK: Natural killer
NSCLC: Non-small cell lung cancer
PD-1: Programmed cell death protein 1
PD-L1: Programmed death-ligand 1
RT: Radiotherapy
SCNA: Somatic copy number aberration
SNV: Single nucleotide variant
TILs: Tumour infiltrating lymphocytes
TMB: Tumour mutational burden
TME: Tumour microenvironment
Tregs: Regulatory T cells
UVB: Ultraviolet B
